# Antioxidant Activity and Hepatoprotective Potential of *Polyalthia longifolia* and *Cassia spectabilis* Leaves against Paracetamol-Induced Liver Injury

**DOI:** 10.1155/2012/561284

**Published:** 2012-11-08

**Authors:** Subramanion L. Jothy, Azlan Aziz, Yeng Chen, Sreenivasan Sasidharan

**Affiliations:** ^1^Institute for Research in Molecular Medicine (INFORMM), Universiti Sains Malaysia (USM), Gelugor, 11800 Penang, Malaysia; ^2^Dental Research & Training Unit and Oral Cancer Research and Coordinating Centre (OCRCC), Faculty of Dentistry, University of Malaya, 50603 Kuala Lumpur, Malaysia

## Abstract

In the present study, *in vitro* antioxidant, free radical scavenging capacity, and hepatoprotective activity of methanol extracts from *Polyalthia longifolia* and *Cassia spectabilis* were evaluated using established *in vitro* models such as ferric-reducing antioxidant power (FRAP), 2,2-diphenyl-1-picryl-hydrazyl (DPPH^•^), hydroxyl radical (OH^•^), nitric oxide radical (NO^•^) scavenging, metal chelating, and antilipidperoxidation activities. Interestingly, all the extracts showed considerable *in vitro* antioxidant and free radical scavenging activities in a dose-dependent manner when compared to the standard antioxidant which verified the presence of strong antioxidant compound in leaf extracts tested. Phenolic and flavonoid content of these extracts is significantly correlated with antioxidant capacity. Since *P. longifolia* extract was exhibited better *in vitro* antioxidant activities, it was subjected for *in vivo* hepatoprotective activity in paracetamol-intoxicated mice. Therapy of *P. longifolia* showed the liver protective effect on biochemical and histopathological alterations. Moreover, histological studies also supported the biochemical finding, that is, the maximum improvement in the histoarchitecture of the liver. Results revealed that *P. longifolia* leaf extract could protect the liver against paracetamol-induced oxidative damage by possibly increasing the antioxidant protection mechanism in mice. Our findings indicated that *P. longifolia* and *C. spectabilis* have potential as good sources of natural antioxidant/antiaging compounds.

## 1. Introduction 

Oxidation process is essential to many living organisms for the production of energy to fuel biological processes. However, oxygen-centered free radicals and other reactive oxygen species (ROS) which are continuously produced *in vivo* may result in cell death and tissue damage. The free radicals are fundamental in modulating various biochemical processes and represent an essential part of aerobic life and metabolism [1]. The most common ROS include superoxide anion (O_2_^−^), hydrogen peroxide (H_2_O_2_), and hydroxyl radicals (OH^−^) which resulted from cellular redox processes. At low or moderate concentrations, ROS exert beneficial effects on cellular response and immune function but at high levels, these radicals become toxic and disrupt the antioxidant defense system of the body, which may lead to “oxidative stress” [2]. The oxidative stress cascade initiated by free radicals obtains stability through electron pairing with biological macromolecules such as proteins, lipids, and DNA in healthy human cells and causes damage in cell structures that include proteins and DNA along with lipid peroxidation. Moreover, the formation of free radicals shortens cells life span and produces changes that resemble aging. This has contributed to a wide range of diseases including coronary heart diseases, aging, neurodegenerative disorders, arthritis, diabetes, inflammation, lung damage, and cancer [3]. Although aging is likely to be a multifactorial process and not reducible to any one single cause but the evidence from the free radical theory detailing the impact of these free radicals on aging [4]. Since the formation of free radicals has a great impact on the aging process, it is appropriate to examine the role of natural antioxidant as a defense system. Accordingly, plant-based natural antioxidants with free radical scavenging activity are emerging as the primary components of holistic approaches in impeding aging. Therefore, this study focuses on local medicinal plants as alternative nutraceutical sources in impeding aging, namely, *Polyalthia longifolia* and *Cassia spectabilis* (Leguminosae).

Plants are humans' oldest friends and our life, directly or indirectly, depends on various plant-related products in many fields including medicine. Hence, natural products which are disease-free agents are gifts from mother nature. A full range of plant derived nutritional supplements, phytochemicals, and provitamins that help in sustaining good health and fighting diseases is now being described as functional foods, nutriceuticals, and nutraceuticals [5]. Medicinal plants from the tropical and subtropical climates are recognized to possess many medicinal properties. Apart from the commonly consumed vegetables, some underconsumed medicinal plants are known in traditional medicine, especially in rural communities. These are rarely consumed, unknown, and unfamiliar and have not received much attention as antioxidant sources compared to common vegetables. This could be due to their lack of popularity among local people, lack of scientific information on their nutritional values, and lack of promotion or campaigns for these medicinal plants. These medicinal plants are of great importance to consumers considering the biological health benefits they bring and their role as antiaging agents. 

*Polyalthia longifolia *var. angustifolia Thw. (Annonaceae), is a small medium-sized tree with linear-lanceolate leaves, 1 to 1.5 cm broad, occurring in Sri Lanka and now grown in tropical parts of India along road sides and in gardens for their beautiful appearance [6]. *P. longifolia* is one of the most important indigenous medicinal plants and found throughout Malaysia and widely used in traditional medicine as febrifuge and tonic [7]. The diterpenes, alkaloids, steroid and miscellaneous lactones were isolated from its bark [7]. The stem bark extracts and isolated compounds were studied for various biological activities like antibacterial, cytotoxicity and antifungal activity [8]. On the other hand, *Cassia spectabilis* (Leguminosae) is widely grown as an ornamental plant in tropical and subtropical areas and has been commonly used in traditional medicine for many years. It has been used in traditional Brazilian medicine for the treatment of flu, cold and also as a laxative and purgative [9]. The *C. spectabilis* leaf extract is reported to possess various types of pharmacological activities such as antioxidant, antifungal and antimicrobial activities [10, 11]. Therefore, by focusing on these two plants, namely, *P. longifolia* and *C. spectabilis *to determine their antioxidant and free radical scavenging properties, the study further contributes to the knowledge of Malaysian traditional plants. In the present study, an attempt was made to find the relationship between phenolic content and antioxidant activity of these two plants. The function of natural antioxidants as antiaging agents were also correlated and discussed. 

## 2. Materials and Methods

### 2.1. Plant Collection

The leaves of *P. longifolia* and *C. spectabilis *were collected from various areas in Universiti Sains Malaysia, Penang in January 2012 and was identified by Mr Shunmugam Vellosamy at the Herbarium of School of Biological Sciences, Universiti Sains Malaysia, Pulau Pinang, Malaysia where the voucher specimen was deposited (Number: USM/HERBARIUM/11306 for *P. longifolia *and USM/HERBARIUM/11033 for *C. spectabilis*). The leaves, which were separated and cut into small pieces, were first washed with tap water and then with distilled water. They were then dried in an oven at 60°C for 7 days, after which the dried leaves were ground into fine powder using a grinder and stored in clean, labeled airtight bottles.

### 2.2. Solvent Extraction

Dried sample (approximately 100 g) was added to methanol (300 mL) and soaked for 4 days at room temperature (30 ± 2°C). The suspension was stirred from time to time to allow the leaf powder to fully dissolve in the methanol. Removal of the sample from the solvents was done by filtration through cheesecloth followed by filter paper (Whatman no. 1); the filtrate was concentrated under vacuum to one-fifth its volume using a rotary evaporator at 60°C and then sterilized by filtration using a 0.22-mm membrane. The thick paste obtained was further dried in an oven at 40°C. The resultant extract was kept at 4°C for further analysis. Methanol was used for the extraction in this study to mimic the use of water by traditional healers to make decoction. Water and methanol have the highest polarity in the polar protic solvent group. Moreover, the use of methanol makes the process of evaporation easier compared to that of water.

### 2.3. Determination of Total Phenolic and Flavonoid Contents

 A total phenolic content was determined by the Folin-Ciocalteau method [12, 13] with a slight modification. The extract samples (0.1 mL of different dilutions) were mixed with Folin Ciocalteu reagent (0.75 mL, 1 : 10 diluted with distilled water) and incubated for 5 min at 22°C. Then Na_2_CO_3_ (0.5 mL, 7%) was added to the mixture. This mixture was allowed to stand for 90 min at 22°C and the phenols were determined by colorimetric method at 765 nm. The standard curve was prepared by 0, 50, 100, 150, 200, 250, and 500 mg/mL solutions of Gallic acid in methanol : water (50 : 50, v/v). Total phenol values are expressed in terms of Gallic acid equivalent (mg/g of dry mass), which is a standard reference compound. 

Total flavonoid content of the extracts was determined according to colorimetric method described by Zhishen et al. [14] with some modification. Briefly 0.5 mL of extracts (1 mg/mL) were added in three bijoux bottles and mixed with 2 mL of distilled water. Subsequently 0.15 mL of sodium nitrite (NaNO_2_, 5% w/v) was added into each bottle and the reaction mixture was allowed to stand for 6 min. Then 0.15 mL aluminium trichloride (AlCl_3_, 10%) was added and allowed to stand for 6 min, followed by the addition of 2 mL of sodium hydroxide (NaOH, 4% w/v) to the reaction mixture. Distilled water was next added to the mixture to bring the final volume up to 5 mL. The reaction mixture was mixed thoroughly and allowed to stand for another 15 min. The absorbance of pink colour that developed was measured at 510 nm using a spectrophotometer (HITACHI U-1900 spectrophotometer). Distilled water was used as blank. The final absorbance of each sample was compared to a standard curve plotted using catechin. The total flavonoid content was expressed in mg of catechin per gram of extract. All tests were conducted in triplicate.

### 2.4. In Vitro Antioxidant Assays

#### 2.4.1. DPPH-Radical-Scavenging Assay

The stable 1,1-diphenyl-2-picryl hydrazyl radical (DPPH) was used in the determination of free radical scavenging activity of the extract [15]. The reaction mixture contained 50 *μ*L of different concentrations of the extracts and 5 mL of 0.04% (w/v) solution of DPPH in 80% methanol. After 30 min incubation at room temperature, the absorbance was recorded at 517 nm using a spectrophotometer (HITACHI U-1900 spectrophotometer 200 V). The commercially known antioxidant, butylated hydroxytoluene (BHT) was used as a positive control. The experiment was performed in triplicate. The percentage of the DPPH free radical was calculated using the following equation:

(1) $$\begin{array}{c} \text{DPPH scavenging effect}\;\left(\%\right)=\left[\frac{A_{0}-A_{1}}{A_{0}}\right]\times 100, \end{array}$$

where *A*_0_ was the absorbance of the control, and *A*_1_ was the absorbance in the presence of the extract or positive control. The IC_50_ (concentration providing 50% inhibition) values were calculated by using the dose inhibition curve in linear range by plotting the extract concentrations versus the corresponding scavenging effect.

#### 2.4.2. Reducing Power Assay

The reducing power of extracts was determined according to the method proposed by Yen and Chen [16] with slight modification. Different concentrations of extracts (0.5 mL) were mixed with phosphate buffer (2.5 mL, 0.2 M, pH 6.6) and potassium ferricyanide [K_3_Fe(CN)_6_] (2.5 mL, 1%). The mixture was incubated at 50°C for 20 min. A portion (2.5 mL) of trichloroacetic acid (10%) was added to the mixture to terminate the reaction, which was then centrifuged at 3000 rpm for 10 min. The upper layer of solution (2.5 mL) was mixed with distilled water (2.5 mL) and FeCl_3_ (0.5 mL, 0.1%). The reaction mixture was then incubated for 10 min at room temperature and the absorbance was measured at 700 nm. All tests were performed six times. A higher absorbance of the reaction mixture indicated greater reducing power. BHT was used as a positive control.

#### 2.4.3. Hydroxyl Radical Scavenging Assay

The capacity to scavenge hydroxyl radicals was measured according to the method proposed by Halliwell et al. [17] with modification. The hydroxyl radicals are generated by iron-ascorbate-EDTA-H_2_O_2_, which then react with deoxyribose to form thiobarbituric acid reactive substances (TBARS). This substances yield pink chromogen at low pH while heating with trichloroacetic acid (TBA). The hydroxyl scavengers from the leaves extract compete with deoxyribose for hydroxyl radicals and decrease TBARS, which leads to a reduction in the formation of pink chromogen [18]. The reaction mixture contained 4 mM deoxyribose, 0.3 mM ferric chloride, 0.2 mM EDTA, 0.2 mM ascorbic acid, 2 mM H_2_O_2_ and various concentrations of extracts. The tubes were capped tightly and incubated for 30 min at 37°C. Then 0.4 mL of 5% TBA and 0.4 mL of 1% TBA were added to the reaction mixture which was kept in a boiling water bath for 20 min. The intensity of pink chromogen was measured spectrophotometrically at 532 nm against the blank sample. Ascorbic acid was used as a positive control. All tests were performed in triplicate. The hydroxyl radical scavenging activity of leaves extract was reported as % inhibition of deoxyribose degradation and calculated using the following equation:

(2) $$\begin{array}{c} \%\;\text{Inhibition}=\left[\frac{A_{0}-A_{1}}{A_{0}}\right]\times 100, \end{array}$$

where *A*_0_ was the absorbance of the control and *A*_1_ was the absorbance in the presence of the extract or positive control.

#### 2.4.4. Nitric Oxide Scavenging Assay

At physiological pH, nitric oxide generated from aqueous sodium nitroprusside (SNP) solution interacts with oxygen to produce nitrite ions which were measured by the Griess Illosvoy reaction [19]. The reaction mixture contained SNP (10 mM), phosphate buffer saline (pH 7.4) and different concentrations of the extract and incubated for 180 min at 25°C. Then Griess reagent (1% sulphanilamide, 0.1% naphthylethylenediamine dichloride (NED) and 3% phosphoric acid) was added and the mixture was further incubated for 30 min at 25°C. The pink chromophore formed during diazotization of nitrite ions with sulphanilamide and subsequent coupling with NED was measured spectrophotometrically at 540 nm against blank sample. Quercetin was used as a positive control [20]. All tests were performed in triplicate. The nitric oxide radicals scavenging activity was calculated according to the following equation:

(3) $$\begin{array}{c} \%\;\text{Inhibition}=\left[\frac{A_{0}-A_{1}}{A_{0}}\right]\times 100, \end{array}$$

where *A*_0_ was the absorbance of the control, and *A*_1_ was the absorbance in the presence of the extract or positive control. 

#### 2.4.5. Ferrous Ion Chelating Assay

The chelating activity of the extracts for ferrous ions Fe^2+^ was measured according to the Dinis et al. [21] method. Briefly, 0.5 mL of different concentrations of the extract was added to a solution of 2 mM FeCl_2_ (0.05 mL). The reaction was initiated by the addition of 5 mM Ferrozine (0.2 mL). The mixture was shaken vigorously and left at room temperature for 10 min. Ferrozine reacted with the divalent iron to form stable magenta complex species that were very soluble in water. Absorbance of the solution was then measured spectrophotometrically at 562 nm. The percentage inhibition of ferrozine- Fe^2+^ complex formation by leaves extract was calculated as following:

(4) $$\begin{array}{c} \text{Percentage of inhibition}\;\left(\%\right)=\left[\frac{A_{0}-\;A_{1}}{A_{0}}\right]\times 100, \end{array}$$

where *A*_0_ was the absorbance of the control, and *A*_1_ was the absorbance of the extract or ascorbic acid (positive control).

#### 2.4.6. Antilipid peroxidation (ALP) Assays

The antilipid peroxidation activity of methanolic leaves extract of *P. longifolia* and *C. spectabilis* was determined by the standard method followed by the slight modification with the chicken liver homogenate [22]. Chicken liver was obtained from a local market in Penang Island and perfused with normal saline through hepatic portal vein. The liver was harvested and its lobes were briefly dried between filter papers to remove excess blood and cut thinly with a heavy-duty blade. The small pieces were then transferred to a sterile vessel containing phosphate buffer (pH 7.4) solution. After draining the buffer solution as completely as possible, the liver was immediately ground to make a tissue homogenate (1 g/mL) using freshly prepared phosphate buffer (pH 7.4). The extracts with different concentrations (10–0.15625 mg/mL) were mixed with liver homogenate (2.9 mL), 50 mM FeSO_4_ (0.2 mL) and incubated for 30 minutes at 37°C. One mL of reaction mixture was mixed with 2.5 mL of 10% TCA- 0.67% TBA (thiobarbituric acid) in acetic acid (50%) for blocking the reaction. Then the mixture was boiled for 1 hour at 90°C and centrifuged at 10,000 rpm for 5 minutes. Supernatant was taken for absorbance at 535 nm. ALP % was calculated using the following formula:

(5) $$\begin{array}{c} \text{Percentage of inhibition}\;\left(\%\right)=\left[\frac{A_{0}-\;A_{1}}{A_{0}}\right]\times 100, \end{array}$$

where *A*_0_ was the absorbance of the control, and *A*_1_ was the absorbance of the extract or Ascorbic acid (positive control).

### 2.5. *In Vivo* Hepatoprotective Activity of *P. longifolia* Leaf Extract

Since* P. longifolia* showed better antioxidant activity compared with *C. spectabilis* it was further evaluated for *in vivo* hepatoprotective Activity.

#### 2.5.1. Animals

Eighteen specific pathogen-free and age-matched (7- to 10-week-old) male Wister albino mice were used to study the hepatoprotective activity of the *P. longifolia *leaf extract. The Institution Animal Ethics Committee of University has approved the animal study (USM/Animal Ethics Approval/2011/(74) (365)) for this project. The animals were kept at 27 ± 2°C, relative humidity 44–56% and light and dark cycles of 10 and 14 h, respectively, for a week before and during the experiments. Animals were provided with a standard diet (Lipton, India) and water *ad libitum*. The food was withdrawn 18–24 h before starting the experiment. All experiments were performed in the morning according to current guidelines for the care of the laboratory animals and the ethical guidelines for the investigation of experimental pain in conscious animals [23].

#### 2.5.2. Paracetamol Dose Regimen

Paracetamol tablets were obtained from a nearby clinic. Each tablet contains 500 mg of paracetamol. The dose administered to the mice to induce hepatotoxicity was set as 1 g/kg. The paracetamol was turned into a fine powder using a mortar and pestle to increase the dissolution. The powdered paracetamol was suspended in saline and was administered orally according to the body weight of mice.

#### 2.5.3. Grouping of Mice and Treatments

Eighteen mice (25–30 g) were randomly divided into three groups, each group consisting of six mice. The first group received a single daily dose of 1 mL/kg of saline orally (control group). Group II was given a single daily dose of paracetamol (1.0 g/kg) orally (induced group) and Group III received orally a single daily dose of both 1.0 g/kg paracetamol [24] and 200 mg/kg of *P. longifolia* leaf extract, respectively (treated group). *P. longifolia* leaf extract was administered three hours after the administration of paracetamol. The treatments were continued for seven days and on the eighth day of the experiment, all the mice were anesthetized and dissected [25].

#### 2.5.4. Sacrifice and Organ Harvesting

The liver was removed carefully after euthanizing and killing the animals by cervical dislocation. The livers were fixed in 10% buffered formalin. After fixation, the livers were dehydrated in a graded series of alcohol, cleared in xylene and embedded in paraffin wax. Multiple 5 *μ*m sections from each block were mounted on slides and stained with hematoxylin and eosin.

#### 2.5.5. Biochemical Parameters

The mice of each group were anaesthetized with ether, and blood was collected directly from the heart. It was centrifuged at 2,000 g for 10 min at 4°C to separate the serum and kept at 4°C to assay the activities of serum enzymes. Aspartate aminotransferase (AST) and alanine aminotransferase (ALT) were determined by the method described by Reitman and Frankel [26]. Serum bilirubin level was estimated according to Malloy and Evelyn's method [27].

#### 2.5.6. Statistical Analysis

All values are mean ± S.E.M. obtained from six mice in each group. For statistical analysis, one-way ANOVA with Duncan's variance (SPSS 15) was used to compare the groups. In all the cases a difference was considered significant when *P* < 0.05.

## 3. Results and Discussion

### 3.1. Antioxidant Activity

In the present study, *in vitro* antioxidant and free radical scavenging capacity of methanol extracts from *P. longifolia* and *C. spectabilis *methanolic extract were evaluated using established *in vitro* models such as ferric reducing antioxidant power, DPPH, hydroxyl radical, nitric oxide radical, scavenging, and metal chelating activities. It is believed that the role of antioxidant in disease prevention is due to its ability to scavenge free radicals in the biological systems. Antioxidants can deactivate radicals by three major mechanisms: hydrogen atom transfer (HAT), electron transfer (ET), and combination of both HAT and ET [28]. HAT measures the ability of an antioxidant to quench free radicals by hydrogen donation, while ET detects the ability of antioxidant to transfer one electron to reduce radicals, metals and carbonyls [29]. Ferric reducing antioxidant power is an ET assay. DPPH assays combine both HAT and ET mechanisms. 

### 3.2. Total Phenolic and Flavonoid Contents

Antioxidant activity of plants might be due to their phenolic compounds. The highest antioxidant activity reported for the tested extract might be due to the presence of these bioactive constituents. The antioxidant activity of phenolic compounds is mainly due to their redox properties, which can play an important role in adsorbing and neutralizing free radicals, quenching singlet and triplet oxygen, or decomposing peroxides [30]. Crude extracts of medicinal plant materials rich in phenolics are increasingly of interest in the food industry. Therefore, the total phenolic content of *P. longifolia* and *C. spectabilis *methanolic extracts was determined with the Folin-Ciocalteu reagent in this study. Gallic acid was used as a standard compound and the total phenols were expressed as mg/g gallic acid equivalent using the standard curve equation: *y* = 0.0036*x* + 0.3742, *R* ^2^ = 0.8581, where *y* is absorbance at 760 nm, and *x* is total phenolic content in the different extracts of *P. longifolia* and *C. spectabilis* expressed in mg/g extract. *P. longifolia* leaf had the highest TPC (87.43 ± 1.230 mg GAE/g extract) compared to *C. spectabilis* leaf (44.30 ± 2.101 mg GAE/g extarct). Although some studies have demonstrated a correlation between phenolic content and antioxidant capacity [31], our results are in agreement with many other findings. Li et al. [32] stated that no correlation between total phenolic contents and antioxidant activities in jujube fruits is possible because the antioxidant activity observed was not solely from the phenolic contents of jujube. Instead, a substantial fraction of total antioxidant activity could also be due to the presence of other phytochemicals such as ascorbic acid, tocopherol and pigments as well as the presence of synergistic effects among compounds that contribute to the total antioxidant activity. Therefore, these antioxidant compounds may regenerate, increasing their antioxidant activity. Hence, the antioxidant activity observed in this study might also be contributed by various phytochemicals besides phenolic compounds, thus warrants further studies.

Beside phenolic compounds, the presence of flavonoids in leaf extracts might affect the observed antioxidant activity. The flavonoids content obtained from leaf extracts was 70.25 ± 3.12 mg and 35.41 ± 2.82 mg of catechin equivalent (CE) per gram of *P. longifolia* and *C. spectabilis* extracts respectively, with reference to standard curve (*y* = 0.0071*x* + 0.1139 and *r* ^2^ = 0.9927). Flavonoids are a broad class of low molecular weight, secondary plant phenolics characterized by the flavan nucleus. The protective effects of flavonoids in biological systems are ascribed to their capacity to transfer electrons to free radicals, chelate metal catalysts [33], activate antioxidant enzymes [34], reduce alpha-tocopherol radicals [35], and inhibit oxidases [36]. Moreover, the highest total flavonoids amount was found in the *P. longifolia* extract. Based on the antioxidant assays, it is thus suggested that flavonoids present in *P. longifolia* extract have strong antioxidant activities. This could be due to the antioxidant mechanisms of flavonoids towards free radicals.

### 3.3. DPPH Radical Scavenging Assay

**Figure 1:**
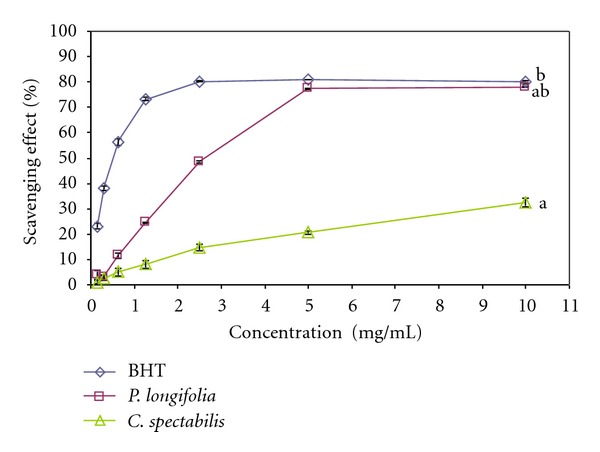
Scavenging effect of methanolic leaf extract of *Polyalthia longifolia* and *Cassia spectabilis* on DPPH free radicals compared with butylated hydroxytoluene (BHT). Each value is expressed as means ± SEM (*n* = 3), (*P* < 0.05).

 The results of the DPPH scavenging activity of the extracts are as shown in Figure 1. The methanolic extracts of *P. longifolia* and *C. spectabilis* leaf exhibited concentration dependent antiradical activity by inhibiting DPPH radical with inhibitory concentration 50% (IC_50_) values of 2.721 ± 0.116 mg/mL (*P. longifolia*), 30.178 ± 0.129 mg/mL (*C. spectabilis*) and while those of the standard was 0.547 ± 0.088 mg/mL (BHT). As a rapid and simple measure of antioxidant activity, the DPPH radical scavenging capacity has been used in this study to determine the free radical scavenging activity of *P. longifolia* and *C. spectabilis* leaf extracts. The DPPH assay was based on the reduction of the stable radical DPPH to yellow colored diphenyl picrylhydrazine in the presence of a hydrogen donor [37]. The results of this study indicate that all the samples tested have noticeable effect on DPPH radical. *P. longifolia* and *C. spectabilis* exhibited sustainable hydrogen donating and radical scavenging abilities. However, *C. spectabilis *exhibited moderate DPPH scavenging activity compared to the *P. longifolia* extract. The extracts of *P. longifolia* and *C. spectabilis* showed lower DPPH scavenging activity compared to the positive control BHT used in this study. It has been reported in literature that the antioxidant activity of many medicinal plants is proportional to their phenolic content, suggesting a contributory relationship between total phenolic content and antioxidant activity [38]. The character of phenolics contributing to their electron transfer/hydrogen donating ability is also reported to be associated to the DPPH radical scavenging activity [39]. Radical scavenging activity is very important due to the deleterious role of free radicals biological systems. *P. longifolia* and *C. spectabilis* have demonstrated good free radical scavenging activity which ensured the ability to reduce the harmful free radicals in the maintenance of health and the management of aging.

### 3.4. Reducing Power Assay

**Figure 2:**
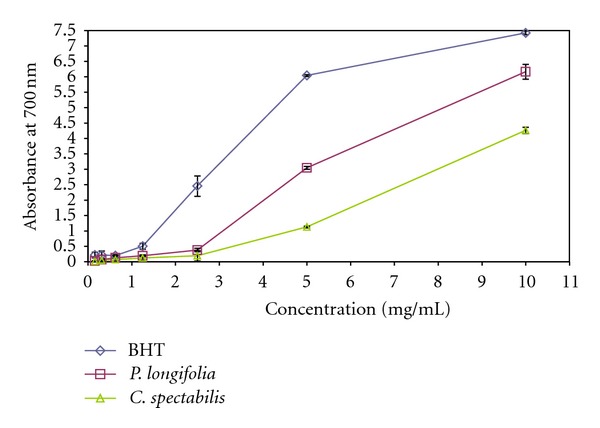
Reducing power of methanolic leaf extract of *Polyalthia longifolia* and *Cassia spectabilis* compared to butylated hydroxytoluene (BHT). Each value is expressed as means ± SEM (*n* = 3).

As shown in Figure 2, *P. longifolia* and *C. spectabilis* leave extracts exhibited increased ferric reducing power with increased concentration. The reducing power of extracts and standard antioxidants decreased in the order of BHT >*P. longifolia* >*C. spectabilis*. In reducing power assay, the presence of antioxidants in the samples would result in reducing Fe^3+^ to Fe^2+^ by donating an electron by the extract. The extract with reducing power reveal that they are electron donors, reduce the oxidized intermediates and act as primary antioxidant substances [40]. In this assay, the yellow colour of the test solution changes to various shades of green and blue depending on the reducing power of each compound present in the leave extracts. The compounds with reduction potential react with potassium ferricyanide (Fe^3+^) to form potassium ferrocyanides (Fe^2+^), which then react with ferric chloride to form ferric ferrous complex that is greenish in colour.

(6) Potassium ferricyanide+Ferric chloride  →Potassium ferrocyanides+ferrous chloride.

The amount of Fe^2+^ complex was monitored by measuring the formation of Perl's Prussian blue at 700 nm. Increasing absorbance at 700 nm indicated an increase in reductive ability. The higher absorbance of extract may be due to its strong reduction potential. The reducing power of the extracts may be caused by the bioactive compounds in the extracts which possess potent donating abilities. This assay further confirmed the antioxidant properties of the extracts observed from the DPPH assay. The correlation between reducing power and DPPH values could be due to the same mechanism on which these methods rely. The results clearly indicated that the extracts tested can act as electron donors and react with free radicals, and convert them to more stable products, thus terminating the radical chain reaction and preventing aging related problem.

### 3.5. Hydroxyl Radical Scavenging Assay

**Figure 3:**
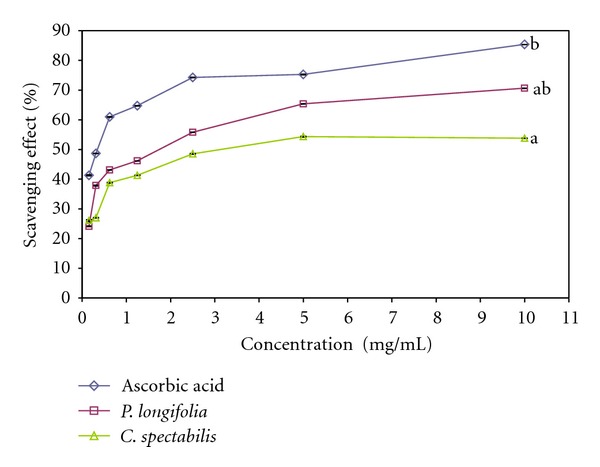
Scavenging effect of methanolic leaf extract of *Polyalthia longifolia* and *Cassia spectabilis* on hydroxyl radicals compared to ascorbic acid. Each value is expressed as mean ± SEM (*n* = 3), (*P* < 0.05).

The Hydroxyl radical is the most reactive oxygen species that induces severe damage in biomolecules. The hydroxyl scavenging ability of methanolic extracts of *P. longifolia* and *C. spectabilis* was estimated by generating hydroxyl radicals using deoxyribose method [17]. The hydroxyl radical generated through fenton reaction which degraded deoxyribose using Fe^2+^ as an important catalytic component. The potential of leaf extract to inhibit hydroxyl radical-mediated deoxyribose damage was determined by means of iron (II) dependent DNA damage assay. In this assay, the test and standard compound colour changed to various shades of pink. Antioxidant efficiency of the leave extracts compared to the standard control ascorbic acid was determined as the ability to scavenge the free radicals generated. The results are shown in Figure 3. The IC_50_ values of *C. spectabilis* leave extract were less than those of *P. longifolia* extract which were 1.377 ± 0.083 and 3.874 ± 0.081 mg/mL, respectively. While the standard ascorbic acid shows good antioxidant activity with IC_50_ values of 0.315 ± 0.085 mg/mL to scavenge 50% of hydroxyl radicals. The ability of the extracts to quench hydroxyl radicals can be related to the prevention of lipid peroxidation. Moreover, it seemed to be a good scavenger of active oxygen species, thus reducing the rate of chain reaction.

### 3.6. Nitric Oxide Scavenging Assay

**Figure 4:**
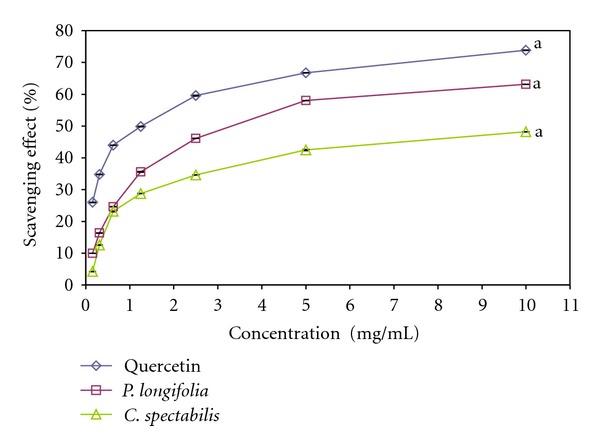
Scavenging effect of methanolic leaves extract of* Polyalthia longifolia* and *Cassia spectabilis* on nitric oxide radicals compared with Quercetin. Each value is expressed as means ± SEM (*n* = 3), (*P* > 0.05).

Nitric oxide (NO) or reactive nitrogen species such as NO_2_, N_2_O_4_, N_3_O_4_, NO_3_, and NO_2_ are formed during the reactions of nitrogen with oxygen or with superoxides which are very reactive. These compounds are responsible for altering the structural and functional behavior of many cellular components. Plant/plants products may have the property to counteract the effect of NO formation and in turn may be of considerable interest in preventing ill effects of excessive NO generation in the human body. NO is also implicated for inflammation, cancer, and other pathological conditions [41]. The relative NO scavenging potential of the extracts of *P. longifolia *and *C. spectabilis* in the study as compared with standard control quercetin are presented in Figure 4. 

In summary, the radical scavenging potential of extracts obtained was in the following order: quercetin >*C. spectabilis *>* P. longifolia* with inhibitory concentration 50% (IC_50_) values of 3.472 ± 0.090 mg/mL (*P. longifolia*), 8.549 ± 0.093 mg/mL (*C. spectabilis*) and while that of the standard was 1.178 ± 0.083 mg/mL (Quercetin). Moreover, both tested leave extracts showed good NO-scavenging activity which has the potential to inhibit the nitric oxide in dose-dependent manner. Due to rich phytochemicals and their aroma, medicinal plants have become a vital source of chemoprotective agents. This research provides comparative information about local medicinal plants with respect to their NO-scavenging activity. According to the findings, *P. longifolia *and *C. spectabilis* exhibited dose-dependent manner of NO-scavenging activity. It means that the use of leaf extracts as an antioxidant healthy beverage as well as the protection against NO-mediated damage and aging is highly promising.

### 3.7. Ferrous Ion Chelating Assay

**Figure 5:**
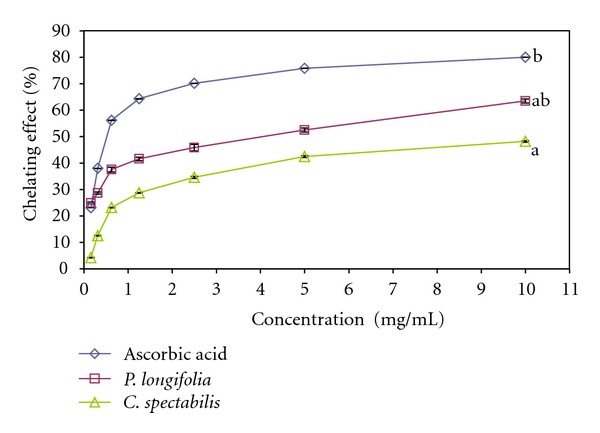
Chelating effect of methanolic leaves extract of *Polyalthia longifolia* and *Cassia spectabilis* on ferrous ions compared to ascorbic acid. Each value is expressed as mean ± SEM (*n* = 3), (*P* < 0.05).

In this assay, the chelating of ferrous ions by the methanolic leaves extracts of *P. longifolia* and *C. spectabilis* was evaluated. Ferrozine produces a violet complex with Fe^2+^. In the presence of other chelating agents, the ferrozine complex formation is interrupted and as a result the violet colour of the complex is decreased.  Figure 5 shows chelating effect of methanolic leave extracts of *P. longifolia* and *C. spectabilis* on ferrous ions compared to that on ascorbic acid. This assay demonstrated that the formation of ferrozine-Fe^2+^ complex was interrupted at high concentrations of test and standard solution, which resulted in a reduction in violet colour complex. This may indicate the absorbance of ferrozine-Fe^2+^ complex and decreased dose-dependence due to the presence of chelating agents. The IC_50_ values of the leave extracts of *P. longifolia *and *C. spectabilis* were 3.079 ± 0.082 mg/mL and 8.549 ± 0.093 mg/mL, respectively. The standard control ascorbic acid showed strong activity and needed 0.666 ± 0.086 mg/mL for 50% inhibition of complex formation. The *P. longifolia* leaf extract revealed good ion chelating activity compared to the *C. spectabilis *extract. The ability of chelating the ferrous ions by the methanolic leaf extract of *P. longifolia* and *C. spectabilis* proved that these antioxidants can deactivate the free radicals by electron transfer to the free radicals. 

### 3.8. Antilipid Peroxidation Activity

**Figure 6:**
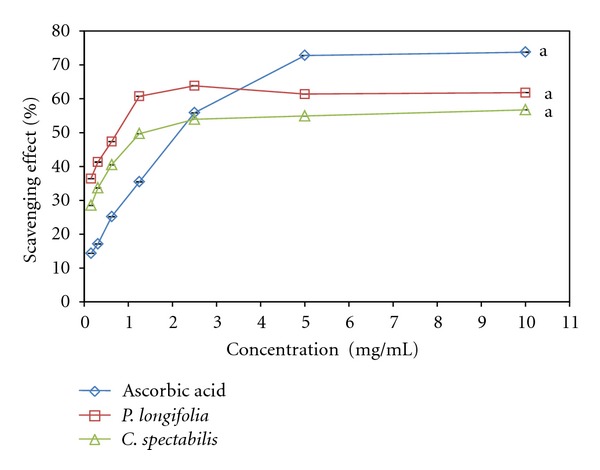
Antilipidperoxidation activity of methanolic leaf extract of *Polyalthia longifolia* and *Cassia spectabilis* compared to ascorbic acid. Each value is expressed as mean ± SEM (*n* = 3), (*P* > 0.05).

In this study, an experiment was performed to analyze the antihyperlipidemic effect of methanolic extract of the selected plants on chicken liver homogenate from 10.000 mg/mL to 0.156 mg/mL by measuring the levels of malondialdehyde (MDA), which were produced, based on the acid-catalyzed decomposition of lipid peroxides. The analysis confirmed that antilipid peroxidation activity (IC_50_) was at 0.774 ± 0.080 mg/mL of *P. longifolia *and at 2.373 ± 0.081 mg/mL of *C. spectabilis *compared to 2.089 ± 0.091 mg/mL of standard ascorbic acid. Antioxidant activity of *P. longifolia, C. spectabilis *and ascorbic acid was gradually increased when the test concentration was increased gradually. From these experimental results, it was certainly confirmed that the methanolic extracts of both plants have potent antioxidant property; with the *P. longifolia *extract having more potent antioxidant property in comparison to the *C. spectabilis *extract and standard ascorbic acid (Figure 6 and IC_50_ value). The antilipid peroxidation assay is a good *in vitro* model for *in vivo* cellular oxidation. Antioxidants may offer resistance against the oxidative stress by scavenging the free radicals and inhibiting the lipid peroxidation and other mechanisms [42]. In conclusion the present study demonstrated that *P. longifolia *and* C. spectabilis* has significant antilipid peroxidative and free radical scavenging activity, which might be useful in preventing or suppressing the progress of different oxidative stress related diseases and aging.

### 3.9. Biochemical Parameters

The effects of *P. longifolia* extract on liver marker enzymes and serum bilirubin content are displayed in Table 1. The data exhibited that the control group demonstrated a normal range of AST, ALT, and bilirubin levels while the paracetamol-treated group showed elevated levels of AST, ALT, and bilirubin, thus confirming that paracetamol causes liver injury at higher doses. The elevation of cytoplasmic AST and ALT is considered an indicator for the release of enzymes from disrupted liver cells. Bilirubin concentration has been used to evaluate chemically induced hepatic injury. Besides its various normal functions, the liver excretes the breakdown product of hemoglobin, namely bilirubin, into bile. It is well known that necrotizing agents like paracetamol produces sufficient injury to the hepatic parenchyma to cause large increases in bilirubin content [43]. Furthermore, the *P. longifolia* extract-treated group showed a very interesting result. Based on the Table 1 data, it is apparent that the biochemical parameters of the extract-treated group are higher than those of the control group (*P* < 0.05), yet show much lower levels of AST, ALT, and bilirubin than the paracetamol-treated group. In other words, the *P. longifolia* extract treatment significantly reduced the previously raised levels of AST, ALT, and bilirubin in hepatotoxic mice. The decrease in the serum levels of these enzymes might be due to the presence of various phenolic compounds in the leaf extract that enhanced the liver's regeneration ability. 

### 3.10. Histopathology Analysis

**Figure 7:**
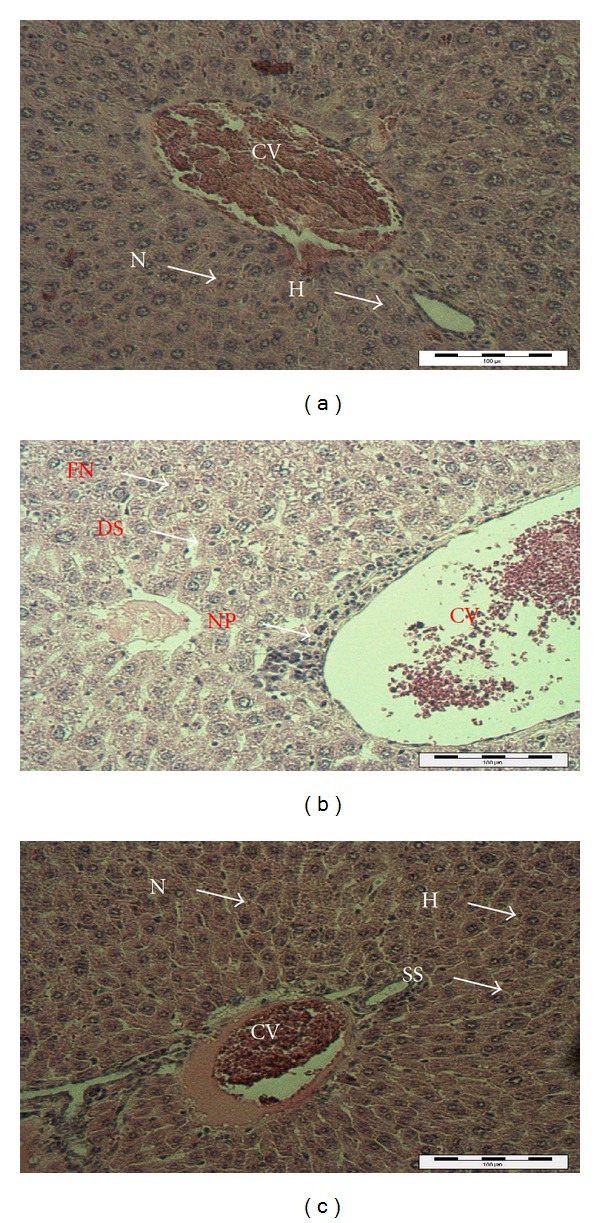
Light microphotographs of liver cell of control (a), mice exposed to paracetamol (b) and treated with *P. longifolia *extract (c). (CV, central vein; H: hepatocytes; N: nucleus; SS: sinusoid; NP: Neutrophil; DS: dilated sinusoid; FN: focal necrosis).

The results of light microscopy examination of the transverse section of control, paracetamol-treated and extract-treated mice livers are presented in Figure 7.  Figure 7(a) shows the liver cells of mice in the control group. From that image, it can be observed that the liver cells are normal in shape with a well-preserved cytoplasm, prominent nucleus and clear central vein (CV). Hepatic cells are arranged in cord like fashion, which are separated by sinusoids. Overall, a healthy set of cells can be observed.  Figure 7(b) shows the liver of paracetamol intoxicated mice showing wide necrosis across the cells. The liver sections of these mice indicate necrosis, ballooning and degeneration in hepatic plates and loss of cellular boundaries. There was also a heavy accumulation of neutrophils surrounding the portal vein. These neutrophils act as an indicator of the occurrence of cell damage as they are absent in normal healthy tissues. The hepatocytes are disrupted and sinusoids (DS) are damaged as well.  Figure 7(c) shows the histological architecture of treated liver sections with a mild degree of degeneration and necrosis. The hepatocytes nucleuses are at a recovery stage and there are very minimal numbers of neutrophils surrounding the portal vein. Hence, therapy with *P. longifolia* extract was effective in restoring the paracetamol induced histopathological lesions and almost normal liver architecture was exhibited, with well-formed hepatocytes separated by sinusoids (SS) and maintained cord arrangement. The histopathological examination thus verified the hepatoprotective effect of *P. longifolia* extract against the model hepatotoxicant paracetamol.

## 4. Conclusions

Collectively, data of this study provide direct evidencethat *P. longifolia *is a potential antiaging candidate, which possesses good *in vivo* hepatoprotective activity. An overdose of paracetamol results in the generation of free radicals following the oxidative stress in the liver cells [44]. However, when the mice were pretreated with *P. longifolia* leaf extract, the biochemical parameters in the liver significantly dropped compared to the group receiving paracetamol only. Obviously, liver cells with numerous mitochondria are more vulnerable to free radical oxidation than any other cells in the body since mitochondria form reactive oxygen species during oxidative phosphorylation. Furthermore, oxidative damage caused by free radicals is arguably the most popular current explanation of aging [45]. The results of this study also showed that the *P. longifolia* possesses antioxidant activity with polyvalent actions towards various free radicals such as superoxide anion, hydrogen peroxide, and hydroxyl radicals, which are commonly found in mitochondria. This model provides a rational explanation for *P. longifolia* leaf extract to be developed as antiaging agents. In conclusion, the present study has demonstrated that *P. longifolia* and *C. spectabilis *leaves possessed good antioxidant activity. The hepatoprotective activity of *P. longifolia* may be due to its free radical-scavenging and antioxidant activity, resulting from the presence of some flavonoids and phenolic compounds in the extracts. The findings thus establish the potential medicinal value of *P. longifolia* and *C. spectabilis *used in traditional systems of medicines and suggest that could be developed as an antiaging agent to determine the lead bioactive compounds.

## Figures and Tables

**Table 1:** Effect of *P. longifolia* leaf extract on liver marker enzymes and serum bilirubin content.

Parameters	Control group	Paracetamol treated group	*P. longifolia* extract treated group
AST (IU/L)	36.72 ± 6.12	111.72 ± 11.28**	46.51 ± 7.11*
ALT (IU/L)	33.42 ± 5.21	93.24 ± 11.26**	38.19 ± 6.87*
Bilirubin (mg/L)	1.7 ± 0.4	7.9 ± 4.1**	3.1 ± 1.4*

Results are expressed as mean ± S.E.M; *Statistically significant compared to paracetamol treated animals (*P* < 0.05); **Statistically significant to control animals (*P* < 0.05).
